# Supplementary material for: Can 3D RVEF be Prognostic for the Non-Ischemic Cardiomyopathy Patient but Not the Ischemic Cardiomyopathy Patient? A Cardiovascular MRI Study
Source: Diagnostics (Basel). 2019 Jan 23;9(1):16. doi: 10.3390/diagnostics9010016 (PMC6468852; doi:10.3390/diagnostics9010016)
Supplement: Supplementary file 1 [file diagnostics-09-00016-s001.pdf]

Table S1: Exclusion Criteria For Our Study Subjects

- Combined ICMX and NICMX
- Ordinal reporting of the RVEF
- LVEF > 35%
- Patients lacking follow-up data for alive-dead status or MACE were excluded from the KM analysis comparing the EF sub groups in ICMX and NICMX patients
